# Warfarin and Antibiotics: Drug Interactions and Clinical Considerations

**DOI:** 10.3390/life13081661

**Published:** 2023-07-30

**Authors:** Alexis J. Vega, Caitlin Smith, Hannah Grace Matejowsky, Katherine J. Thornhill, Grant E. Borne, Chizoba N. Mosieri, Sahar Shekoohi, Elyse M. Cornett, Alan D. Kaye

**Affiliations:** 1 School of Medicine, Louisiana State University Health Sciences Center at Shreveport, 1501 Kings Highway, Shreveport, LA 71103, USA; ajv001@lsuhs.edu (A.J.V.); cms004@lsuhs.edu (C.S.); hgm002@lsuhs.edu (H.G.M.); kjt001@lsuhs.edu (K.J.T.); geb002@lsuhs.edu (G.E.B.); 2 Department of Anesthesiology, Louisiana State University Health Sciences Center at Shreveport, 1501 Kings Highway, Shreveport, LA 71103, USA; chizoba.mosieri@lsuhs.edu (C.N.M.); sahar.shekoohi@lsuhs.edu (S.S.); alan.kaye@lsuhs.edu (A.D.K.); 3 Department of Pharmacology, Louisiana State University Health Sciences Center at Shreveport, 1501 Kings Highway, Shreveport, LA 71103, USA

**Keywords:** warfarin, antibiotics, penicillin, macrolides, fluoroquinolones, cephalosporins, rifampin

## Abstract

Warfarin administration poses a notable challenge in clinical practice due to the increased susceptibility of patients to major bleeding, particularly when co-administered with other medications capable of modulating its metabolic pathways. Among these medications, antibiotics have been recognized as potential agents that can either induce or inhibit cytochrome P450-2C9, thereby impacting the effects of warfarin. A wealth of evidence from numerous studies consistently supports an elevated risk of serious bleeding in patients concurrently receiving antibiotics and warfarin therapy. This narrative review elucidates the intricate interactions between warfarin and various antibiotic classes. Notably, significant increases in the International Normalized Ratio (INR) were observed among warfarin-treated patients receiving penicillin derivatives, fluoroquinolones, TMP-SMX, and macrolides. Conversely, investigations have also demonstrated a reduction in INR levels in patients on warfarin when exposed to rifampin, a potent inducer of cytochrome P-450. Intriguingly, cephalosporin antibiotics and amoxicillin/clavulanate, despite not interfering with the cytochrome P450 system, exhibited a positive association with increased INR values. The findings of this narrative review underscore the importance of diligent monitoring in patients on warfarin requiring concomitant antibiotic therapy, as this surveillance strategy proves pivotal in mitigating the risk of major bleeding complications. Additionally, for patients necessitating cytochrome P450 inhibitors such as penicillin derivatives, fluoroquinolones, TMP-SMX, and macrolides, the consideration of dose reduction in warfarin therapy may confer substantial benefits in reducing the occurrence of major bleeding events. Similarly, patients who are co-administered rifampin alongside warfarin necessitate vigilant monitoring, with a potential need for escalating warfarin doses to counteract the risk of a hypercoagulable state.

## 1. Introduction

Warfarin is the most widely used oral anticoagulant in North America and in the world [1,2]. It has a long-established efficacy for the prevention of thromboembolic events in patients with cardiovascular risk factors such as chronic atrial fibrillation, prosthetic heart valves, venous thromboembolism, and coronary artery disease [1]. Atrial fibrillation is present in >2 million people in the United States and is a common thrombotic cause of ischemic stroke [3]. Valvular heart disease affects 2.5% of the US population and its prevalence is growing worldwide as a consequence of improved survival and the aging population, with many possessing prosthetic heart valves [4]. According to the American Heart Association, on the basis of NHANES 2017 to March 2020 data, the prevalence of cardiovascular disease in adults ≥20 years of age was 48.6% overall (127.9 million in 2020) and increases with age in both males and females. On the basis of 2020 mortality data, heart disease and stroke currently claim more lives each year than cancer and chronic lower respiratory disease combined. In 2020, 207.1 of 100,000 people died of heart disease and stroke [5,6]. These statistics reflect the importance of anticoagulant medications for the prevention of thromboembolic events.

Over the past decade, anticoagulation with dual oral anticoagulants such as dabigatran, rivaroxaban, apixaban, and edoxaban have become more prevalent in the prevention of thromboembolic events in patients with cardiovascular risk factors due to ease of use and lower associated bleeding risks. DOACs have become popular with patients on oral anticoagulants because they do not require frequent international normalized ratio monitoring, clinic visits, and dietary restrictions, unlike warfarin [7]. However, DOACs are not as affordable for lower-income individuals or those whose insurance does not cover the cost. It has been reported that treatment of atrial fibrillation with warfarin is cheaper than DOACs, as DOACs are many times more expensive [8]. Although DOACs have been shown to produce greater quality-adjusted life expectancy than warfarin, they may not represent good value for money; therefore, warfarin management is still very important and highly relevant given its extensive need and greater access to patients of all socioeconomic statuses [5].

Warfarin’s anticoagulant effect is achieved through the inhibition of vitamin K-dependent clotting factors II, VII, IX, and X, which involves competitive blocking of vitamin K epoxide reductase complex 1, an enzyme essential for vitamin K activation [9]. By diminishing the levels of these clotting factors, warfarin effectively reduces clot formation and mitigates the risk of thromboembolic events [9].

Given its narrow therapeutic index, warfarin necessitates frequent monitoring and dose adjustments to maintain the delicate balance between adequate anticoagulation and the risk of bleeding or thrombotic complications. Monitoring is typically conducted by assessing prothrombin time (PT) and the international normalized ratio (INR), with diligent surveillance of any elevations in PT/INR levels to prevent adverse outcomes [9].

However, one common complication associated with warfarin therapy is the heightened risk of major bleeding, particularly when co-administered with medications capable of influencing its metabolism [10]. In particular, antibiotics have the potential to interfere with warfarin’s anticoagulant effect through various mechanisms. Antibiotics can induce or inhibit the activity of cytochrome P450-2C9, an enzyme crucial for warfarin metabolism. Additionally, some antibiotics can disrupt the population of vitamin K-producing bacteria in the intestines, further modulating warfarin’s pharmacological response. As a result, these drug interactions can either enhance or diminish warfarin’s efficacy, with potential clinical consequences.

The importance of managing these interactions becomes evident when considering the narrow therapeutic index of warfarin. Increased warfarin levels can have detrimental effects, while subtherapeutic levels may lead to inadequate anticoagulation. And while some antibiotic classes carry a higher risk of bleeding events than others, a comprehensive understanding of the potential interactions between different antibiotics, including penicillin derivatives, fluoroquinolones, cephalosporins, sulfa drugs, anti-mycobacterial agents, macrolides, and metronidazole and warfarin, is necessary [11].

In addition to assessing the risk of bleeding events associated with specific antibiotic classes, it is also important to consider potential mechanisms underlying these interactions. Understanding the factors contributing to warfarin–antibiotic interactions can provide valuable insights into individualized treatment strategies and guide clinicians in making informed decisions regarding dosing adjustments to minimize the occurrence of major bleeding events.

Overall, this narrative review aims to consolidate and elucidate the existing literature on various antibiotic interactions with warfarin, shedding light on their clinical implications and supporting evidence-based strategies for the safe and effective management of patients receiving concurrent therapy.

## 2. Methods

This is a narrative review. The sources for this review are as follows: searching on PubMed, Google Scholar, Medline, and ScienceDirect; using keywords: warfarin, antibiotics, penicillin, macrolides, fluoroquinolones, cephalosporins, and rifampin. Sources were accessed between February 2023 and July 2023.

## 3. Results

### 3.1. Penicillins

There are four major categories of penicillin drugs, all of which have the same mechanism of action. Penicillin V and G are prototype beta-lactam antibiotics and function by acting as D-Ala-D-Ala structural analogs, binding penicillin-binding proteins and blocking the transpeptidase cross-linking of peptidoglycan within the bacterial cell walls [12]. Another group of penicillin drugs is penicillinase-sensitive: Amoxicillin, Ampicillin, and Aminopenicillin. Likewise, there are a group of penicillinase-resistant penicillin drugs whose structure blocks access of the beta-lactamase to the beta-lactam. Lastly, antipseudomonal penicillin drugs are a special group of piperacillin and ticarcillin [12].

Penicillin drugs most commonly treat Gram-positive pathogens due to their thick cell wall filled with peptidoglycan [12]. Some examples include staphylococcal and streptococcal species. Penicillinase-sensitive penicillins have coverage against *H. influenzae*, *H. pylori*, *E. coli*, *L. monocytogenes*, *P. mirabilis*, *Salmonella*, *Shigella*, and *enterococci* species related to their extended spectrum. Antipseudomonal penicillins have exceptional coverage against Pseudomonas.

In a case review of a 39-year-old man with a history of deep vein thrombosis and septic arthritis, concurrent cefazolin and nafcillin with warfarin was examined [13]. Initially receiving cefazolin, the patient’s INR remained within the reference range. When he was later given nafcillin as a replacement for cefazolin, he experienced extreme INR decline. The study demonstrated that the co-administration of nafcillin with warfarin may require a 2–4 fold-increased dose of warfarin to reach INR levels within the reference range. Hepatic cytochrome-P450 induction via nafcillin and dicloxacillin decreases the efficacy of warfarin by enhancing warfarin’s metabolism using cytochrome-P450 [14].

Some sources have stated that oral amoxicillin has not been shown to interact with warfarin [14]. However, many cases of the co-administration of amoxicillin or amoxicillin/clavulanate and warfarin are reported in the literature demonstrating INR elevation and/or bleeding anywhere between 7 days post-amoxicillin initiation to 9 days after amoxicillin therapy was discontinued [15,16]. In a 2003 case report by Davydof et al., a 58-year-old Hawaiian/Asian/European woman developed microscopic hematuria and an elevated INR 2.5 weeks after stopping antibiotic therapy with amoxicillin/clavulanate [14]. This case report describes the importance of considering other contributing factors when describing warfarin’s interaction with antibiotics, including warfarin’s 30 h half-life, the rate of clotting factor synthesis, and depletion time for vitamin K liver stores. Thus, the onset of coagulopathy can range from 7 days after initiating antibiotics to 4 weeks after discontinuation.

When administered high doses, 85.7% of patients experienced INR levels greater than four in a study by Mahmoud et al. The study reported high doses of amoxicillin/clavulanate are associated with a higher risk of over-anticoagulation when combined with warfarin than when administered in lower doses [17]. This is likely due to most penicillin drugs, other than nafcillin and dicloxacillin, reducing the gut flora, leading to decreased vitamin K-producing bacteria. This manifests as an enhancement of warfarin’s effects and vitamin K deficiency due to reduced vitamin K concentrations [14]. Lower quantities of vitamin K-producing gut bacteria would likely lead to decreased vitamin K-dependent activated clotting factors, enhancing warfarin’s anticoagulant effects.

### 3.2. Fluoroquinolones

Fluoroquinolone antibiotics consist of ciprofloxacin, enoxacin, norfloxacin, ofloxacin, as well as a few respiratory fluoroquinolones, gemifloxacin, levofloxacin, and moxifloxacin. The mechanism of action of fluoroquinolones is via inhibition of the prokaryotic enzymes topoisomerase II and topoisomerase IV [18]. Fluoroquinolones are especially useful in treating urinary tract infections and gastrointestinal and respiratory infections. Fluoroquinolones are commonly used to treat infections with Gram-negative rods. They can also be used for some Gram-positive organisms, Pseudomonas, and otitis externa [18].

Ciprofloxacin and levofloxacin are cytochrome-P450 inhibitors, meaning they displace warfarin from binding sites and prevent warfarin metabolism, thus causing a prolonged bleeding time and increased INR levels [18,19]. These adverse effects are seen commonly in patients receiving chronic warfarin therapy for clotting disorders and are placed on a fluoroquinolone antibiotic to treat an infection. In a case study examining four patients on chronic warfarin therapy with concurrent levofloxacin use, three patients experienced INR increase from within the reference range 2–3 to a 3.5, 8.12, and 11.5 as the study progressed. The fourth patient only experienced mild bleeding throughout the trial [19]. 

In a statistical review of 30 patients who were hospitalized and concurrently taking warfarin and levofloxacin, statistical analysis revealed a significant increase in the INR with a supporting *p*-value of 0.0001 [20].

### 3.3. Cephalosporins

Cephalosporin antibiotics also have a beta-lactam ring, like penicillin drugs. They inhibit cell wall synthesis and are less susceptible to penicillinases [21]. There are five generations of cephalosporin antibiotics. First-generation cephalosporins, including cefazolin and cephalexin, are commonly used during surgery to prevent wound infections. Susceptible organisms include *P. mirabilis*, *E. coli*, and *K. pneumoniae*. Second-generation cephalosporins are cefaclor, cefoxitin, cefuroxime, and cefotetan. These are also used to treat the previously mentioned organisms: *Enterobacter* species, *H. influenzae*, *Neisseria* species, and *Serratia*. Third-generation cephalosporins include ceftriaxone, cefpodoxime, and ceftazidime, which are commonly used for severe Gram-negative infections such as *N. meningitidis* and can cross the blood–brain barrier. Fourth-generation cephalosporins include cefepime which is increasingly used against *Pseudomonas*. Lastly, fifth-generation cephalosporins include ceftaroline, which treats MRSA, unlike the first to fourth generation cephalosporins [21]. 

A case review reports ceftaroline prescribed to an 85-year-old woman with a therapeutic INR level who was hospitalized for cellulitis treatment. After a subsequent hospitalization for shoulder pain, her INR level was above therapeutic [22]. It has been found that cephalosporins interact with warfarin by potentiating the risk of hypoprothrombinemia, inhibiting p-glycoprotein, and altering the gastrointestinal flora [22].

A retrospective chart review was conducted by Saum et al. in which INR increases from the baseline were compared in patients taking chronic warfarin therapy with diagnoses of UTI and were treated with ceftriaxone, a first-generation cephalosporin (cefazolin or cephalexin), penicillin (ampicillin/sulbactam, amoxicillin/clavulanate, piperacillin/tazobactam), or ciprofloxacin in a community teaching hospital between June 2011 and September 2012. The ceftriaxone group was found to have a statistically significant higher peak INR value compared to all other antibiotics that were studied (ceftriaxone: 3.56, first-generation cephalosporins: 2.66, penicillins: 2.98, ciprofloxacin: 2.3; *p* = 0.004), a statistically significant greater extent of change in the INR value (+1.19, +0.66, +0.8, +0.275; *p* = 0.006), and a statistically significant greater percentage change in the INR value when compared to ciprofloxacin (54.4% vs. 12.7%; *p* = 0.037) [23]. Ceftriaxone interacts with warfarin to increase a patient’s INR value more than other commonly administered antibiotics for UTI treatment. It was concluded that first-generation cephalosporins, penicillin, or ciprofloxacin should be preferred for UTI treatment in patients on warfarin [23].

### 3.4. TMP-SMX

TMP-SMX is theorized to interact with warfarin via two mechanisms. The first is the disruption of the gut flora, reducing vitamin K synthesis, a shared mechanism with many other classes of antibiotics. The second interaction mechanism is via the inhibition of cytochrome P450 isozyme 2C9, which is also involved in warfarin metabolism [24]. 

In a study by Fischer et al. investigating the interactions between TMP-SMX and warfarin in an elderly population with urinary tract infections, hospitalized cases of upper GI tract hemorrhage in patients on long-term warfarin therapy had an increased risk of having received a prescription for TMP-SMX within 14 days of hospitalization with an odds ratio of 3.84 (95% CI, 2.33–6.33) [25]. Additionally, TMP-SMX prescription showed an increased risk of upper GI tract hemorrhage in patients taking warfarin with an odds ratio of 2.80 (95% CI, 1.48–5.32) compared with amoxicillin or ampicillin, two medications with no observed significant interaction with warfarin [25]. 

In a study by Vitry et al., patients on chronic warfarin therapy who were administered TMP-SMX were associated with increased bleeding-related hospitalizations with a 5.08 adjusted rate ratio (95% CI, 2.00–12.88) [26].

In another study by Baillargeon et al., TMP-SMX treatment in chronic warfarin users aged 65 years or older had an increased risk of hospitalization for bleeding with an odds ratio of 2.70 (95% CI, 1.46–5.05) [10]. Lane et al. evaluated a population of veterans who were prescribed warfarin for ≥30 days and were found to have an increased risk of serious bleeding events when co-prescribed TMP-SMX with a hazard ratio of 2.09 (95% CI, 1.45–3.02). Additionally, 31% of patients prescribed TMP-SMX were found to have an elevation of INR ≥5 [11]. Schelleman et al. investigated the interaction between TMP-SMX and warfarin, which was observed over time intervals after the administration of TMP-SMX was tracked via hospitalization for GI bleeding [27]. When fully adjusted for confounding variables, 0–5 days had an odds ratio of 1.46 (95% CI, 1.16–1.85); 6–10 days had an odds ratio of 2.54 (95% CI, 2.08–3.10); 11–15 days had an odds ratio of 2.04 (95% CI, 1.64–2.54); and 16–20 days had an odds ratio of 1.18 (95% CI, 0.89–1.57) [24].

### 3.5. Rifampin

Interaction between rifampin and warfarin is due to rifampin’s nonspecific induction activity on hepatic enzymes, including cytochrome P450 enzymes, with CYP3A4 being affected preferentially [27]. The current literature and data shows that it can take up to 2 weeks for rifampin’s full effect on these enzymes. After the discontinuation of rifampin, it can take four weeks or longer for warfarin effects to level out again, though this level may not be the same as before rifampin use [27].

Yang et al. studied patients on prior steady-state warfarin who received rifampin. They were investigated for changes in warfarin dosing over time, including after the discontinuation of rifampin. During the onset phase, defined as rifampin initiation in patients who were previously taking warfarin, the study found a median increase in steady-state warfarin dosing on rifampin by 165% (*n* = 8, IQR 99, 227), which occurred in a median of 30 days (*n* = 8, IQR 19, 34). Although the overall warfarin dosing increased, the study showed a decrease of 38% (IQR −52, 32) in warfarin dosing in the first week of rifampin initiation. During the offset phase, defined as when rifampin was discontinued and warfarin continued, the study found a median decrease in steady-state warfarin dosing by 67% (IQR −70, −58), which took a median of 6 weeks to achieve (IQR 4, 8). Compared to before rifampin use, the steady-state dosing of warfarin after discontinuation increased by a median of 8% (*n* = 8, IQE 1, 38) [27].

### 3.6. Macrolides

**Figure 1:**
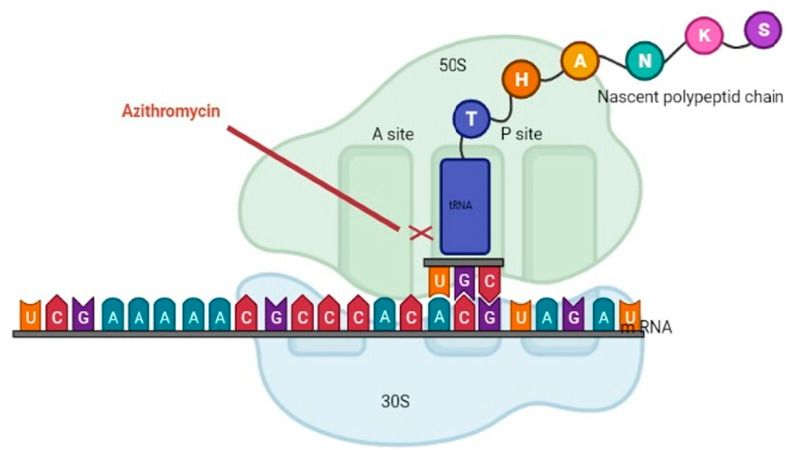
The macrolide family of antibiotics prevents bacterial protein synthesis by binding to the 50 S subunit of the bacterial ribosome. This figure was adapted from Ref. [32] Clinical Laboratory Analysis, Volume: 36, Issue: 6, First published: 21 April 2022.

The macrolide family of antibiotics prevents bacterial protein synthesis by binding to the 50 S subunit of the bacterial ribosome (Figure 1) [28]. Macrolides are considered bacteriostatic because they inhibit protein synthesis without directly killing the bacteria. The bacterial ribosome structure is highly conserved across almost all bacterial species, allowing macrolides to be classified as broad spectrum [29]. The most commonly used macrolides are erythromycin, clarithromycin, azithromycin, and fidaxomicin. Macrolides are indicated in the treatment of *H. pylori*, atypical mycobacterial infections, as well as Gram-positive infections of the skin, respiratory tract, and soft tissue [30].

Extensive research has been conducted regarding the side effects of concomitantly prescribing macrolides alongside warfarin. One study found that macrolide use during warfarin therapy is associated with an increased risk of bleeding with an OR of 1.86, while another study specifically looking at this same interaction in the elderly population found an adjusted risk ratio of 3.07 [26].

Another study looking at azithromycin specifically found it to have a twofold increased risk of a serious bleeding event, compared to low-risk antibiotic usage during warfarin therapy [11].

Other studies also demonstrate an increase in the mean INR from 2.7 to 3.6 when receiving macrolides alongside warfarin treatment in pediatric cardiac patients [31].

### 3.7. Metronidazole

As the drug of choice for Helicobacter, Bacteroides, Clostridia, Giardia, Trichomonas, and Entamoeba species, metronidazole is a broad-spectrum antibiotic known to inhibit anaerobic Gram-negative rods [33]. A member of the nitroimidazole group of drugs, metronidazole is in a class of its own as an antibiotic due to its ability to also treat a variety of parasitic infections. Metronidazole is thought to assert its effects through its reduced intermediates inhibiting the nucleic acid synthesis in specific organisms [34]. 

In a study that appraised over 793 citations relating to food and drug interactions with warfarin, metronidazole was named as one of six antibiotics that potentiate warfarin’s anticoagulant effects [35].

The interactions described in the literature with regard to metronidazole and warfarin are usually found to describe the impact this combination of drugs can have on a patient’s INR. One study found that metronidazole, used alongside warfarin, increased a patient’s INR above 6 in 4.9% of patients [11]. Another study, in a similar vein, described metronidazole use with warfarin as having a 23.3% chance of increasing a patient’s INR into supra-therapeutic levels, which meant an INR greater than 3 [36].

## 4. Discussion

Patients on chronic warfarin therapy have been shown to have an increased INR when prescribed antibiotics that are inhibitors of cytochrome-P450, requiring a lower dose of warfarin to decrease the risk of major bleeding. Pairing warfarin with antibiotics that are cytochrome-P450 inducers has been shown to decrease INR, requiring heavier doses to achieve the protective benefit of anticoagulation. Other factors that may affect warfarin dosing with patients on antibiotics include age and infection status. 

The patients reviewed in our investigation were hospitalized for a variety of reasons, including cellulitis, septic arthritis, and urinary tract infection, and many others were hospitalized for undocumented reasons. Although we did observe a case report of a 39-year-old male and a study regarding pediatric cardiac patients, most of the patients in this research are in the elderly population and veterans, many of whom are in older age groups. 

A retrospective cohort study by Gurwitz et al. in 1992 stated that there were no significant differences in the use of medications that potentiated or inhibited the anticoagulant effects of warfarin across age groups. They also found that when adjusted for dose, the PT ratio was significantly increased in older patients (*p* < 0.001) [37].

In 2003, Froom et al. described the major finding in their study, that even after adjustment for other predictive factors, for every ten year increase in age, there was a 15% increase in the risk of INR values that incurred a temporary cessation of oral anticoagulation therapy [38]. In 2005, a study by Torn et al. observed that the incidence of bleeding and thromboembolic events increase sharply with advanced age [39]. The study results showed the incidence rate of major hemorrhage rose gradually with age from 1.5 per 100 patient-years for patients younger than 60 years to 4.2 per 100 patient-years for patients older than 80 years, yielding a hazard ratio of 2.7 (95% confidence interval, 1.7–4.4). The incidence rate of major thromboembolism rose from 1.0 per 100 patient-years for patients younger than 60 years to 2.4 per 100 patient-years for patients older than 80 years (hazard ratio, 2.2; 95% confidence interval, 1.2–4.2) [39].

A prospective cross-sectional observational study conducted at the Cardiology Department and Intensive Care Unit (ICU) of the Assiut University Hospitals described patients with infection had a higher median discharge of INR than other patients [40]. The patients who participated in the study all received antibiotics. It was concluded that the higher median INR among these patients was probably secondary to the interaction of antibiotics with warfarin. However, a study was conducted on 12,006 patients, which found that acute respiratory tract infection increases the risk of excessive anticoagulation independent of antibiotic use [40]. These considerations may also affect patient management and antibiotic dosing regimens. 

Limitations of our review include the small size of study populations and the lack of randomization observed in many of the studies. For example, race and gender were not considered within the population data, which could have influenced the INR measurement. Another limitation of the literature review is the administered doses were not compared between and within antibiotic classes when examined against fluctuations in the INR. The microbial pathogens treated with the antibiotics were never compared and were not considered confounders in any of the studies. Most of the populations examined within our sources were hospitalized, which could have led to a low level of randomization, as hospitalized patients may have serious conditions that may affect the INR prior to antibiotic administration. Further studies on antibiotics and warfarin co-administration should emphasize significant differences between patient demographics, infection pathogens, and disease severity (Table 1).

## 5. Conclusions

By itself, warfarin carries a risk of serious hemorrhage, so the INR must be monitored frequently in patients who require warfarin for anticoagulation [10]. Therefore, understanding the interaction between warfarin and other drugs, which may enhance warfarin’s bleeding risk or even lead to a hypercoagulable state, is crucial to the field of medicine. Many studies have estimated the number of deaths associated with drug-resistant infections and sepsis and found that infection remains a leading cause of morbidity and mortality world-wide. Hence, infections are commonly seen in hospital settings, and careful consideration of all adverse events and complications should be in place. Studies on warfarin’s interaction with specific antibiotic classes are limited and there are few studies that discuss and compare various antibiotic classes and their interaction with warfarin. Our paper seeks to review the literature on different antimicrobial classes, their interaction with warfarin, and clinical considerations in medical practice.

Most of the antimicrobials in our review are cytochrome-P450 inhibitors, meaning they displace warfarin from binding sites and prevent warfarin metabolism, thus causing prolonged bleeding times and increased INR levels. Precise pharmacological management is important for patient care as an INR greater than 3.5 has been shown to increase the risk of serious bleeding and intracranial hemorrhage. These findings all point to the serious considerations a physician must have when prescribing antibiotics to a patient undergoing warfarin therapy. Physicians should decrease warfarin dosages in cases of patients who are co-prescribed drugs that are cytochrome-P450 inhibitors, such as penicillin derivatives, fluoroquinolones, TMP-SMX, and macrolides. This should also be the case for patients requiring cephalosporins and amoxicillin/clavulanate. These have been shown not to interfere with the cytochrome-P450 system, but are still associated with higher INR levels by altering the gastrointestinal flora [21]. Interaction between rifampin and warfarin is due to rifampin’s nonspecific induction activity on hepatic enzymes, including cytochrome P450 enzymes, with CYP3A4 being affected preferentially. Studies have shown associations between increased warfarin doses for patients who received rifampin. Our review suggests the necessity in de-escalating warfarin therapy when a patient is concomitantly on rifampin [27].

There are many ways to help ensure the proper dosing of warfarin when a patient requires antimicrobial therapy. Technology can be utilized by developing protocols in the electronic medical record (EMR) to flag orders for the above antimicrobials when a patient is already taking warfarin. Perhaps all drugs in the EMR should have a brief description of their mechanism of action, making it more likely to alert providers of possible drug interactions. Warfarin and its various drug interactions should not be missed when prescribing medications, especially antibiotics, in which CYP3A4 inhibitors can enhance the bleeding risk and CYPP 3A inducers can put the patient in a hypercoagulable state.

## Figures and Tables

**Table 1:** Summary of the clinical studies.

Study Name	Year	Type of Study	Participants	Methods	Results	Conclusions
Chaudhuri A, et al. [41]	2018	Retrospective study	Patients who were receiving flucloxacillin/other antibiotics for the minimum of 14 days	Variation in warfarin dose was measured	Patients treated with flucloxacillin had a remarkable enhancement in warfarin dose in the last 7 days of antibiotic therapy. No notable change in warfarin dose for cases on other antibiotics was reported.	International normalized ratio (INR) monitoring is necessary for cases on a continued flucloxacillin treatment.
Mannheimer B, et al. [42]	2019	Retrospective study	Patients who were treated with warfarin	INR values and warfarin doses were measured daily.	For patients with 10 days of treatment, the proportion of cases with a subtherapeutic INR of <2 increased from 22% in the week preceding. In patients with 30 days of treatment, the proportion enhanced from 34 to 63% by 42 days.	INR monitoring is required in patients taking flucloxacillin.
Hellfritzsch M, et al. [43]	2020	Cohort study	212,182 warfarin users	Hazard ratios by evaluating 21-day risks of stroke/ embolism were assessed.	In comparison with phenoxymethylpenicillin, dicloxacillin/flucloxacillin was correlated with hazard ratios of stroke/embolism.	Dicloxacillin needs to be prescribed with caution in cases getting warfarin.

## Data Availability

Not applicable.
